# RAF and antioxidants prevent cell death induction after growth factor abrogation through regulation of Bcl-2 proteins^☆^

**DOI:** 10.1016/j.yexcr.2013.07.029

**Published:** 2013-10-15

**Authors:** Katarzyna Koziel, Julija Smigelskaite, Astrid Drasche, Marion Enthammer, Muhammad Imtiaz Ashraf, Sana Khalid, Jakob Troppmair

**Affiliations:** Daniel Swarovski Research Laboratory, Department of Visceral-, Transplant- and Thoracic Surgery, Innsbruck Medical University, Austria

**Keywords:** RAF, Antioxidants, Reactive oxygen species (ROS), Mitochondria, Bcl-2 proteins, Apoptosis

## Abstract

We have shown previously that mitochondrial ROS production is essential to turn growth factor (GF) removal into cell death. Activated RAF, AKT, Bcl-2 and antioxidants protected equally well against ROS accumulation and subsequent death. Here we investigated whether protection by survival signaling and antioxidants utilizes shared or distinct targets. Using serum deprivation from NIH 3T3 fibroblasts and IL-3 withdrawal from promyeloid 32D cells, we showed that pro-survival signaling by activated RAF but not AKT prevented the decline in Mcl-1 following GF abrogation. GF starvation increased levels of Bim in both model systems, which was prevented by RAF in 32D cells but not in NIH 3T3 fibroblasts. RAF and AKT suppressed activation and mitochondrial translocation of BAX. Also, antioxidant treatment efficiently prevented BAX activation and death of 32D cells but showed little effect on its mitochondrial translocation. No significant impact of antioxidant treatment on Bim or Mcl-1 expression was observed. ROS produced during GF abrogation also did not alter the activity of intracellular signaling pathways, which have been implicated previously in cell killing by pro-oxidants. Together these data suggest Bcl-2 family proteins as convergence point for RAF and ROS in life and death decisions.

## Highlights

• RAF and antioxidants show equal protection against ROS and cell death.
• Antioxidants prevented BAX activation but not mitochondrial translocation.
• No significant impact of antioxidants on Bim or Mcl-1 expression was observed.
• ROS did not alter the activity of intracellular signaling pathways.
• Bcl-2 proteins are critical for the survival activity of RAF and antioxidants.

## Introduction

Stress to cells causes production of reactive oxygen species (ROS), which are causally linked to functional impairment and finally cell death. ROS are key factors in the development of many diseases and pathological conditions ranging from cancer to ischemia–reperfusion injury [1]. While mitochondrial permeabilization, release of cytochrome *c* and ultimately caspase activation and cell death are usually the endpoint in the response to cellular stress, less clear is the nature of events, which initially commit the cell to death under these conditions [2]. Growth factor (GF) abrogation provides a simple and elegant model to study processes involved in life–death decisions and to test intervention strategies. While our work suggested the increase in mitochondrial ROS levels as a key event in cell death commitment after GF removal [3], others identified the degradation of the prosurvival protein Mcl-1 following phosphorylation by GSK3 as an essential step during this time period [4]. Our experiments also demonstrated that increasing mitochondrial Ca^2+^ levels was critical for killing of cells by ROS [3]. Both oncogenic and wild type C- and B-RAF were able to suppress deregulation of mitochondrial homeostasis [3]. Apoptosis regulation by RAF is complex and also has been linked to the upregulation of pro-survival proteins, the inactivation of pro-apoptotic proteins and the recruitment of various effectors including PI3K/AKT and NF-κB [5]. The antioxidant effect of RAF signaling was also confirmed in melanoma cells carrying a mutant form of B-RAF, which responded to MEK inhibition with increased ROS production, which sensitized the cells to killing by BH3 mimetics [6].

Pro-apoptotic effects of ROS may directly damage biomolecules while lower levels modulate intracellular signaling [1]. Redox stress also triggers the activation of the intrinsic cell death pathway. Both, BAX and BAK and an increase in mitochondrial Ca^2+^ were required for ROS-induced cell death in MEFs [7]. In our model the use of the antioxidant *N*-acetyl-cysteine (NAC) proved equally efficient in protecting GF-deprived cells from apoptotic cell death as activated RAF, AKT or the pro-survival protein Bcl-2 [3]. The effects of ROS on signaling and cell death most frequently have been studied by exogenously adding pro-oxidants to cells and rarely by following up endogenously produced ROS. The goal of the work presented here was to (i) further study the effect of RAF signaling in preventing apoptotic cell death and excessive mitochondrial ROS levels after GF removal, and (ii) to identify events during the activation of the intrinsic cell death pathway, which are subject to the regulation by ROS. Two cellular models were used in our experiments, 32D cells and NIH 3T3 cells expressing various forms of activated RAF. The data presented identify members of the Bcl-2 family as targets for regulation by RAF and antioxidants. Our work also shows that endogenously produced ROS have only limited effects on the activation of intracellular signaling pathways implicated in the regulation of cell death or survival. Finally, we also find no evidence that the maintenance of low endogenous ROS levels by RAF occurs via effects on the cellular antioxidant capacity suggesting that RAF signaling may connect to mitochondrial ROS production.

## Material and methods

### Reagents and antibodies

RAF kinase inhibitor BAY43-9006 (sorafenib, 1397) was purchased from Axon Medchem BV, Groningen, The Netherlands, the MEK inhibitor UO126 (V1121) from Promega Corporation, Madison, USA, and the PI-3 kinase inhibitor LY294002 (440202) from Calbiochem, Darmstadt, Germany. *N*-acetyl-cysteine (NAC) and 4-hydroxytamoxifen (OHT) were obtained from Sigma Aldrich, Dorset, UK. OHT was dissolved in ethanol as solvent, which also was used as a control in cell assays. Primary antibodies against PARP (9542), caspase-3 (9662), Bim (2819), phospho-AKT Ser473 (4058), AKT (4685), phospho-GSK-3α/β Ser21/9 (9331), GSK-3 (9338), BAX (2772) and phospho-p38 Thr180/Tyr182 (9211) were purchased from Cell Signaling Technology, Boston, MA, antibodies recognizing phospho-ERK Thr202/Tyr204 (sc-16982), ERK (sc-94), p38 (sc-535), JNK (sc-571) and VDAC (sc-8828) were obtained from Santa Cruz Biotechnology, Santa Cruz, CA, Mcl-1 (600-401-394) from Rockland Immunochemicals, Gibertsville, PA, phospho-JNK (AF1205) from R&D systems, Minneapolis, MN, α-tubulin (T5168) from Sigma Aldrich, Dorset, UK and GAPDH (AM4300) from Ambion, Grand Island, NY.

### Cell culture and viral infections

Mouse fibroblast cell lines NIH 3T3 WT and NIH 3T3 expressing constitutively active forms of RAF kinases EHneo/gag-v-RAF [8], C-RAF S427G [9] or B-RAF V600E [10] (kindly provided by R. Marais, The Patterson Institute for Cancer Research, The University of Manchester, Manchester, UK) were grown in Dulbecco's modified Eagle's medium (DMEM) (PAA Laboratories, Pasching, Austria) containing 10% fetal calf serum (FCS) (PAA Laboratories, Pasching, Austria), 200 mM L-glutamine and penicillin (100 U/ml) streptomycin (100 µg/ml) (PAA Laboratories, Pasching, Austria). Promyeloid interleukin-3 (IL-3)-dependent 32D WT cells were cultivated in RPMI medium (PAA Laboratories, Pasching, Austria) containing 10% WEHI 3B conditioned medium as source of IL-3, 10% (FCS) (PAA Laboratories, Pasching, Austria), 200 mM L-glutamine, penicillin (100 U/ml) streptomycin (100 µg/ml) (PAA Laboratories, Pasching, Austria).

To generate NIH 3T3 cells carrying active form of AKT and NIH 3T3 or 32D cells expressing 4-hydroxytamoxifen (OHT)-inducible mutant of C-RAF, ERBXB (11) cells were infected with retroviruses expressing activated AKT/PKB, pBABE-puro AKTα m/p [11] (kindly provided by B. Hemmings, FMI, Basel) or pBABE-puro ERBXB [12] (kindly provided by E. Kerkhoff), respectively. Production of retroviruses and retroviral infection were performed as described before [13]. In order to induce ERBXB expression in NIH 3T3 and 32D cells, cells were cultivated in growth or starvation media in the presence of solvent (ethanol) or 200 nM OHT.

### GF starvation of cells

To induce starvation of NIH 3T3 fibroblasts, cells were washed 2 times with serum free DMEM and incubated in DMEM containing 0.05% FCS for 12 h. 32D cells were washed 3 times with IL-3 free RPMI containing 10% FCS, resuspended in IL-3 free 10% FCS RPMI and incubated for 16 or 24 h. In the experiments employing application of kinase inhibitors NIH 3T3 cells were restimulated with 10% serum for 10 min followed by cell collection.

### Immunobloting

Cells were starved for the indicated times and collected using trypsin (Sigma Aldrich, Dorset, UK). Cells lysis was performed in NP-40 buffer (25 mM TRIZMA base, 150 mM NaCl, 10 mM Na_4_P_2_O_7_, 25 mM β-glycero-phosphate, 10% glycerol, 0.75% NP-40, 25 mM NaF, pH 7.2) containing 1:100 protease inhibitor cocktail set I (Calbiochem, Darmstadt, Germany). Protein concentration was determined by using Bio-Rad DC protein assay kit (Bio-Rad, Hercules, CA, USA). 20 µg of protein was separated on 10% or 12% polyacrylamide gel and transferred to a nitrocellulose membrane (Whatman Inc., Dassel, Germany). After blocking the membrane with 5% skim milk powder (Fluka, Buchs, Switzerland) dissolved in TBST (50 mM TRIZMA base, 150 mM NaCl, pH 7.5 adjusted with HCl, 0.1% Tween-20). The membrane was incubated overnight with appropriate primary antibody prepared according to the protocol provided by manufacturer. After 3 washing steps with TBST the membrane was further incubated for 1 h in HRP-conjugated secondary antibody, diluted in TBST with 5% skim milk powder, washed with TBST and developed using ECL substrates (Thermo Fisher Scientific Inc, Rockford, IL).

### BAX immunoprecipitation

3×10^6^ NIH 3T3 or 10×10^6^ 32D cells (500,000 cells/ml) were seeded on 10 cm^2^ tissue culture plate. After starvation NIH 3T3 cells were lysed for 30 min in ice-cold CHAPS buffer (150 mM NaCl, 10 mM HEPES, 1% CHAPS, pH 7.4) containing 1:100 protease inhibitor cocktail set I (Calbiochem, Darmstadt, Germany) and collected using the cell scraper. 32D cells were pelleted, washed once with PBS (PAA Laboratories, Pasching, Austria), resuspended in ice-cold CHAPS buffer and kept on ice for 30 min. The cells were further broken up by passing NIH 3T3 cells through 25G and 32D cells through 27G needles for six times. The lysate was spun down at 16,000*g* for 10 min at 4 °C and protein concentration was determined. 650 µg lysate protein were incubated with 2 µg of 6A7 BAX antibody (556467, BD Pharmingen) shaking overnight at 4 °C. The remaining lysate was used as full lysate control. Protein G Agarose (Roche Diagnostic, Wien, Austria) was added and the sample was shaken for the next 5 h at 4 °C. The agarose beads were washed 3 times with ice-cold CHAPS buffer, combined with Laemmli sample buffer [14] and boiled at 95 °C for 5 min. The equal volume of samples was used for immunobloting analysis with anti-BAX antibody (2772, Cell Signaling).

### Mitochondria isolation

To isolate mitochondria 3×10^6^ NIH 3T3 cells or 10–15×10^6^ 32D cells were seeded on 10 cm tissue culture dish. After starvation NIH 3T3 cells were collected in the isolation buffer (250 mM saccharose, 10 mM Tris, 0.1 mM EGTA, pH 7.4) using the cell scraper and spun down for 5 min at 600*g* at 4 °C. 32D cells were pelleted and washed once with PBS. Cells were then resuspended in isolation buffer and transferred to 3 ml glass homogenizer (Sartorius Mechatronics, Vienna, Austria). Samples were next homogenized on ice, NIH 3T3 with 40 and 32D cells with 60 strokes and spun down for 10 min at 600*g* at 4 °C. To pellet mitochondrial fraction the collected supernatant was centrifuged for 10 min at 7000*g* at 4 °C. Mitochondria were washed 3 times with isolation buffer, resuspended in NP-40 buffer and boiled with sample buffer at 95 °C for 5 min.

### Total antioxidant capacity

NIH 3T3 and 32D cells, cultivated in full growth medium, were lysed in NP-40 buffer (25 mM TRIZMA base, 150 mM NaCl, 10 mM Na_4_P_2_O_7_, 25 mM β-glycero-phosphate, 10% glycerol, 0.75% NP-40, 25 mM NaF, pH 7.2) containing 1:100 protease inhibitor cocktail set I (Calbiochem, Darmstadt, Germany). Protein concentration was determined by using a Bio-Rad DC protein assay kit (Bio-Rad, Hercules, CA, USA). 1 ml of lysate at 1 µg/µl protein concentration was transferred to quartz cuvette with magnetic stirrer and placed in a Schimadzu RF-5301PC spectrofluorophotometer. 2′,7′-dichlorofluorescein diacetate (DCF-DA, Sigma Aldrich, Dorset, UK) fluorescent probe was added to obtain 20 µM final concentration. After addition of hydrogen peroxide (H_2_O_2_, Sigma Aldrich, Dorset, UK) to 20 mM final concentration changes in the fluorescence intensity were monitored using Hyper RF software (Schimadzu, Duisburg, Germany). Data are presented as a mean value of fluorescence increase per minute.

### Statistics

All data are presented here as means±standard deviations (SD). Statistical analysis was performed by using student *t* test for comparison between 2 groups. To calculate statistical differences of multiple groups 2 ways ANOVA followed by the Benferroni post hoc test was performed. Statistical significance was considered at a *p* value of ≤0.05.

## Results

### RAF-controlled steps in the activation of intrinsic cell death pathway

**Fig. 1:**
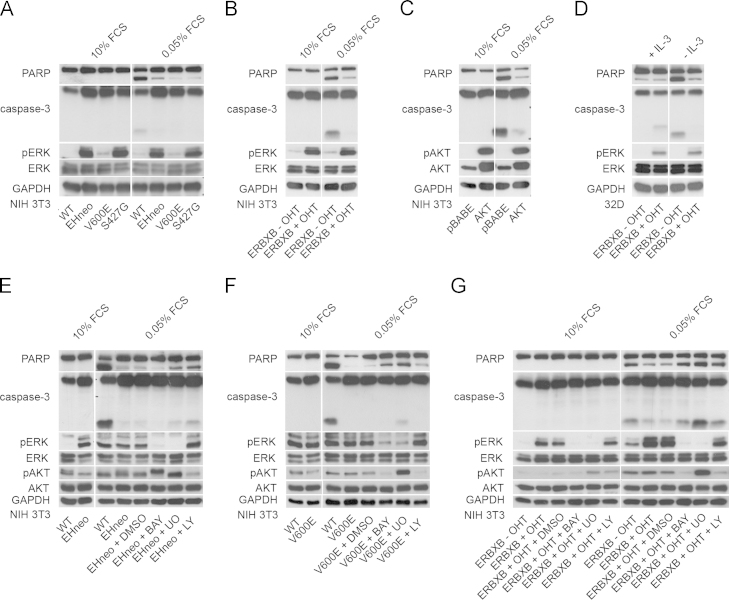
Oncogenic RAF protects against apoptotic cell death (A–D). (A) NIH 3T3 WT, NIH 3T3 gag-v-RAF (EHneo), NIH 3T3 B-RAF V600E (V600E), NIH 3T3 C-RAF S427G (S427G), (B) NIH 3T3 ERBXB (ERBXB) cultured in the presence of 200 nM OHT (+OHT) or its solvent (−OHT), (C) NIH 3T3 pBABE, NIH 3T3 pBABE AKTm/p (AKT) cells were incubated in 10% or 0.05% FCS media for 12 h prior to lysis and further analysed. (D) 32D ERBXB cells (ERBXB) (+/− OHT) were cultivated in the presence or absence of IL-3 for 16 h. Protection requires RAF kinase activity, PI3K and MEK (E–G). To assess the requirement of MEK and PI3K (E) NIH 3T3 EHneo, (F) NIH 3T3 V600E and (G) NIH 3T3 ERBXB fibroblasts (+/− OHT) were maintained in 0.05% FCS medium containing either DMSO (solvent), 20 µM BAY43-9006 (BAY), 25 µM UO126 (UO) or 25 µM LY294002 (LY) for 12 h. At the end of the treatment period cells were lysed and analyzed for the activation of RAF and apoptosis signaling using antibodies directed against PARP, caspase-3 (casp3), pERK/ERK and pAKT/AKT. GAPDH protein levels were used as a loading control.

To study effects of RAF/MEK on the activation of the intrinsic cell death pathway, various versions of mutant RAF were tested including the previously studied viral oncogene [3,8,15], the most frequently occurring V600E mutation in human B-RAF [16] and the only bona fide oncogenic human C-RAF mutant isolated from a *t*-AML patient, C-RAF S427G [9], all of them expressed in NIH 3T3 fibroblasts. We chose this cell model because it allowed us to study the effects of mutant RAF in an otherwise untransformed background. NIH 3T3 cells responded to GF abrogation with increased ROS production. However, the use of antioxidants failed to delay cell death (data not shown), while oncogenic B- and C-RAF clearly were cytoprotective. These observations suggest that the amount of ROS produced was insufficient to trigger cell death in NIH 3T3 cells. This cell model therefore allowed us to study survival signaling by RAF in a setting where ROS are not involved in cell death induction. We also included 32D and NIH 3T3 cells expressing an OHT-regulated fusion protein of the hormone-binding domain of the estrogen receptor with the kinase domain of C-RAF, referred to as ERBXB [17,18]. Additionally, a comparison was made with the survival kinase AKT [4]. Apoptosis was induced in fibroblasts by switching cells to medium containing 0.05% serum or by extensively washing out WEHI 3B-conditioned medium, which was used as source of IL-3, from 32D cells. All analyses shown were performed after NIH 3T3 and 32D cells had been maintained under these conditions for 12 h and 16 h, respectively. Cell death was analyzed by AnnexinV/PI (data not shown) staining and at the molecular level by monitoring the processing of the effector caspase-3 (casp3) and the proteolytic cleavage of the caspase substrate poly-ADP ribose polymerase (PARP). All versions of oncogenic RAF efficiently delayed the onset of apoptotic cell death in NIH 3T3 and 32D cells (Fig. 1). In agreement with previously published data [15], inhibiting RAF/MEK impaired RAF survival activity (Fig. 1E–G).

**Fig. 2:**
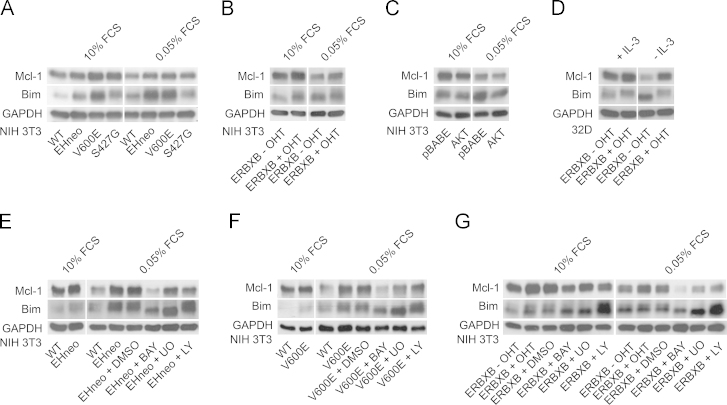
RAF/MEK signaling in the regulation of Mcl-1/Bim expression. Changes in Mcl-1 and Bim protein levels in NIH 3T3 and 32D cells under conditions of limited GF were studied using the following cell models and conditions: (A) NIH 3T3 WT, NIH 3T3 gag-v-RAF (EHneo), NIH 3T3 B-RAF V600E (V600E), NIH 3T3 C-RAF S427G (S427G) (B) NIH 3T3 ERBXB (ERBXB) cultured in the presence of 200 nM OHT (+OHT) or its solvent (−OHT) and (C) NIH 3T3 pBABE, NIH 3T3 pBABE AKTm/p (AKT). Cells were incubated in 10% or 0.05% FCS media for 12 h, (D) 32D ERBXB cells (+/−OHT) were cultivated in the presence or absence of IL-3 for 16 h. To check for the requirement of MEK or PI3K (E) NIH 3T3 EHneo, (F) NIH 3T3 V600E and (G) NIH 3T3 ERBXB fibroblasts (+/− OHT) were maintained in 0.05% FCS medium containing either DMSO (solvent), 20 µM BAY43-9006 (BAY), 25 µM UO126 (UO) or 25 µM LY294002 (LY) for 12 h. These treatments were followed by cell lysis and immunobloting using Mcl-1 and Bim antibodies. GAPDH protein levels were used as a loading control.

In NIH 3T3 cells expressing oncogenic RAF (Fig. 2A) or NIH 3T3 and 32D cells expressing OHT-inducible form of RAF (Fig. 2B, D) Mcl-1 expression levels were higher in cells growing in the presence of 10% serum or 10% WEHI 3B-conditioned medium compared to wild type cells. In both cell models GF removal resulted in the degradation of Mcl-1, which was significantly less pronounced in cells expressing activated RAF (Fig. 2A, B, and D), but not in cells expressing activated AKT (Fig. 2C). These findings demonstrate that Mcl-1 is stabilized in cells expressing oncogenic RAF. Inclusion of RAF/MEK or PI3K kinase inhibitors reversed the RAF effect on Mcl-1 stability (Fig. 2E–G).

Basal Bim levels were increased in cells harboring activated RAF but not AKT in the presence of GF (Fig. 2A–D). Serum starvation of NIH 3T3 cells resulted in a modest increase in Bim expression, which was not prevented by RAF. Additionally, activation of RAF caused a retarded migration of Bim. The requirement of RAF/MEK but not AKT signaling for this modification was shown through the use of inhibitors (Fig. 2E–G). Expression of activated AKT failed to prevent the decrease in Mcl-1 protein following serum removal and the increase in Bim was less pronounced (Fig. 2C). Bim levels significantly increased following GF deprivation in 32D cells (Fig. 2D). Expression of activated RAF prevented the increase of Bim expression following IL-3 deprivation in 32D cells (Fig. 2D). We also tested the effect of inhibiting either RAF/MEK or PI3K signaling in cells expressing activated RAF (Fig. 2E–G). Blocking RAF kinase activity through the application of BAY43-9006 (BAY) in GF starved cells resulted in a dramatic loss of Mcl-1 levels and a concordant drop in Bim levels as well as the change in its electrophoretic mobility. MEK inhibition had a moderate effect on Mcl-1 and only affected Bim mobility but not protein levels. Inhibiting PI3K decreased Mcl-1 expression in NIH EHneo cells (Fig. 2E), but showed no effect on Bim expression or mobility. Similar effects were observed in fibroblasts expressing B-RAF V600E (Fig. 2F) or ERBXB (Fig. 2G).

### Regulation of BAX activation and mitochondrial translocation by RAF signaling

**Fig. 3:**
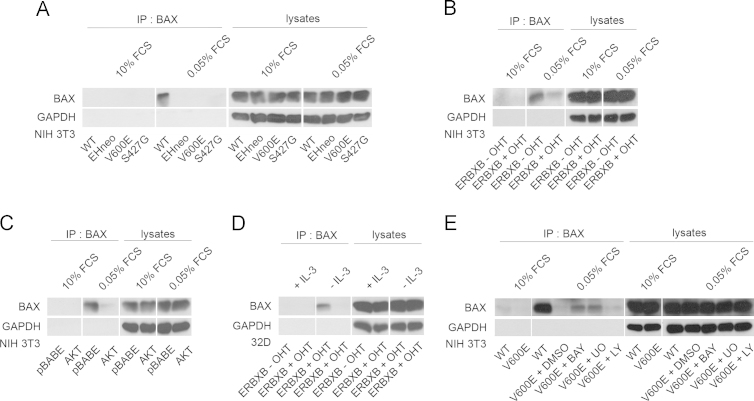
RAF/MEK signaling in BAX activation. BAX activation was anayzed in NIH 3T3 and 32D cells upon GF withdrawal. (A) NIH 3T3 WT, NIH 3T3 gag-v-RAF (EHneo), NIH 3T3 B-RAF V600E (V600E), NIH 3T3 C-RAF S427G (S427G), (B) NIH 3T3 ERBXB cultivated in the presence of 200 nM OHT (+OHT) or its solvent (−OHT), (C) NIH 3T3 pBABE, NIH 3T3 pBABE AKTm/p (AKT) cells were incubated in 10% or 0.05% FCS media for 12 h, (D) 32D ERBXB cells (+/− OHT) were cultivated in the presence or absence of IL-3 for 16 h (E) NIH 3T3 V600E cells were maintained in 0.05% medium containing either DMSO (solvent), 20 µM BAY43-9006 (BAY), 25 µM UO126 (UO) or 25 µM LY294002 (LY) for 12 h, followed by immunoprecipitation (IP) with specific BAX antibody (clone 6A7).

**Fig. 4:**
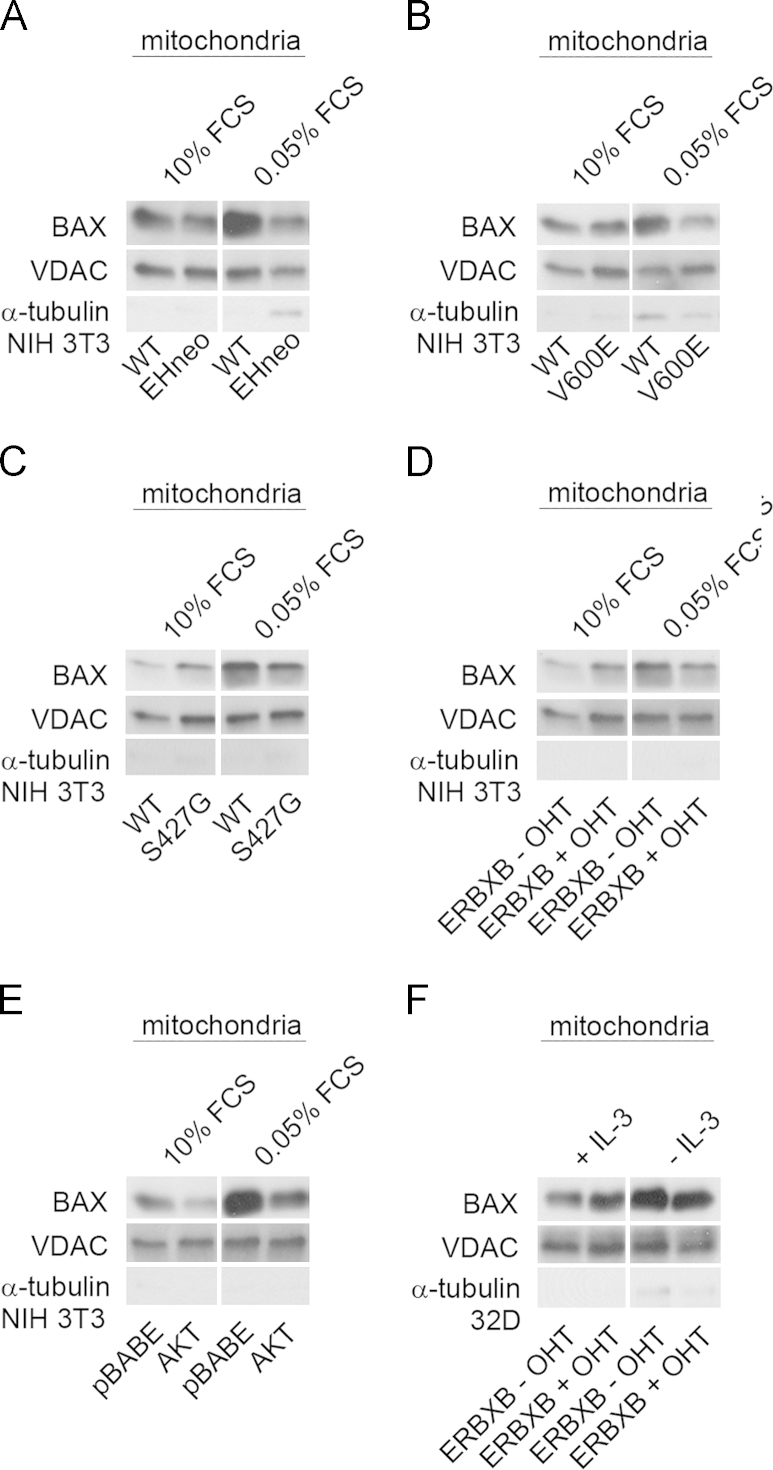
Regulation of BAX translocation to the mitochondria. Changes in the mitochondrial levels of BAX in NIH 3T3 and 32D cells exposed to GF abrogation were analyzed following the isolation of mitochondrial fractions of NIH 3T3 WT, (A) NIH gag-v-RAF (EHneo), (B) NIH 3T3 B-RAF V600E (V600E), (C) NIH 3T3 C-RAF S427G (S427G), (D) NIH 3T3 ERBXB cultured in the presence of 200 nM OHT (+/− OHT), (E) NIH 3T3 pBABE, NIH 3T3 pBABE AKTm/p (AKT) cells maintained in 10% or 0.05% FCS medium for 12 h and (F) 32D ERBXB cells (+/− OHT) cultivated in the presence or absence of IL-3 for 16 h were isolated and analyzed for BAX protein level. Examination of α-tubulin and VDAC served as a confirmation for purity of isolated mitochondria.

Lysates were prepared from 32D and NIH 3T3 cells undergoing GF abrogation and activated BAX was immunoprecipitated with the conformation-specific antibody 6A7 and detected by the total BAX antibody. As shown in Fig. 3, presence of oncogenic RAF (Fig. 3A, B, and D) or AKT (Fig. 3C) prevented BAX activation with similar efficiency. In cells expressing oncogenic B-RAF V600E we also tested the effects of inhibiting RAF, MEK or PI3K (Fig. 3E). Inhibition of B-RAF or MEK partially reverted BAX activation, while PI3K inhibition had no effect. This finding is striking since the application of the inhibitor in this setting completely blocked the phosphorylation of MEK/ERK and suggests kinase-independent functions for RAF in this context. Following activation BAX translocates to the mitochondria. We therefore performed cell fractionation experiment and checked for the presence of BAX in the mitochondrial fraction. As shown in Fig. 4A–D expression of activated RAF and to a lesser degree of AKT prevented the translocation of BAX to the mitochondria following GF removal. Slightly increased mitochondrial BAX levels were observed in some of the cell lines expressing activated RAF.

### Effects of RAF on mitochondrial membrane potential

**Fig. 5:**
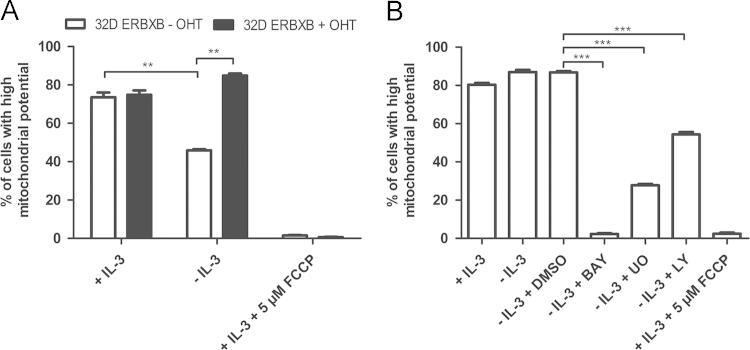
Effects of RAF on mitochondrial membrane potential. The effects of RAF signaling on the mitochondrial membrane potential following GF abrogation were studied in 32D cells expressing an OHT-regulated form of oncogenic RAF after IL-3 removal. (A) 32D ERBXB cells were cultivated in the medium containing 200 nM OHT (+OHT) or solvent (−OHT) in the presence or absence of IL-3 for 24 h and (B) 32D ERBXB cells (±OHT) incubated in IL-3 free medium containing DMSO, 20 µM BAY43-9006 (BAY), 25 µM UO126 (UO) or 25 µM LY294002 (LY) were subjected to mitochondrial membrane potential analysis with FACS. As a positive control for mitochondrial potential dissipation cells were treated with 5 µM FCCP. The graph depicts the percentage of cells gated with high mitochondrial potential (*n*=3, **p≤0.01, ***p≤0.001).

Mitochondrial permeability transition constitutes an important step in life and death decisions. As shown in Fig. 5 activation of RAF was sufficient to prevent the collapse of the membrane potential in 32D ERBXB cells (Fig. 5A). Treatment with the uncoupling agent FCCP served as positive control causing complete collapse of barrier function. The protective effect of RAF again depended on its kinase activity as well as on its downstream effector MEK (Fig. 5B). Again inhibiting PI3K also impaired RAF function.

### Identification of steps in the intrinsic cell death pathway and in intracellular signaling susceptible to the regulation by ROS

**Fig. 6:**
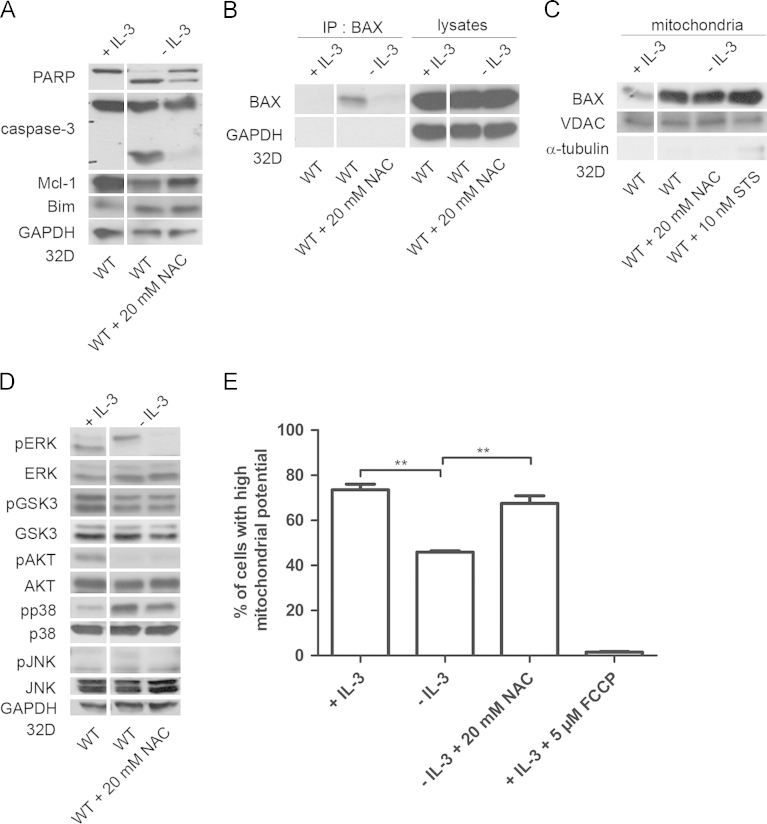
Regulation of intracellular signaling and cell survival by anti-oxidants. 32D cells were starved in the presence of antioxidant *N*-acetyl-cysteine (NAC). 32D WT cells were incubated in full growth medium, IL-3 free medium and in IL-3 free medium containing 20 mM NAC for 16 h, followed by (A) PARP and caspase-3 (casp3) cleavage, Mcl-1 and Bim protein expression, (B) BAX activation detection, (C) BAX mitochondrial protein level examination, (D) intracellular signaling pathways analysis (E) mitochondrial membrane potential was assessed in 32D ERBXB cultured in the presence of 200 nM OHT (+OHT) or its solvent (−OHT) and maintained for 24 h in media supplemented with IL-3, deprived of IL-3, or deprived of IL-3 containing 20 mM NAC (n=3, **p≤0.01).

Apoptosis induction by ROS has been studied primarily in settings, where cells were exposed to exogenously applied pro-oxidants or agents, which caused excessive ROS production. In the model of GF abrogation ROS production occurs as a cellular response to the lack of cellular survival signaling. To study ROS regulation 32D cells were treated with antioxidant that was present during GF abrogation. The antioxidant *N*-acetyl-cysteine (NAC) treatment prevented caspase-3 and PARP processing (Fig. 6A). Antioxidant treatment also prevented BAX activation in 32D cells under the same stress conditions (Fig. 6B), but had little effect on BAX translocation to the mitochondria (Fig. 6C). Pro-oxidant treatment of 32D cells growing in full medium efficiently caused apoptosis (data not shown). No significant changes in the expression of Bim or Mcl-1 were observed.

ROS or ROS generating drugs frequently elicit cell death by activating stress kinase pathways or negatively modulating cell survival pathways. We thus also checked for the involvement of key MAPKs and of the PI3K/AKT signaling under cellular stress and monitored the effects of pro- and anti-oxidants on their activity/activation. Overall, there are only very limited effects of the pro-oxidant treatment or the use of antioxidants on intracellular signaling (Fig. 6D). Thus in conclusion we find no evidence that ROS effects are mediated through effects on the activity of MAPKs pathways. Antioxidant treatment also protected cells from the dissipation of the mitochondrial membrane potential (Δ*Ψ*) caused by GF withdrawal (Fig. 6E).

### Regulation of total antioxidant capacity by RAF signaling

**Fig. 7:**
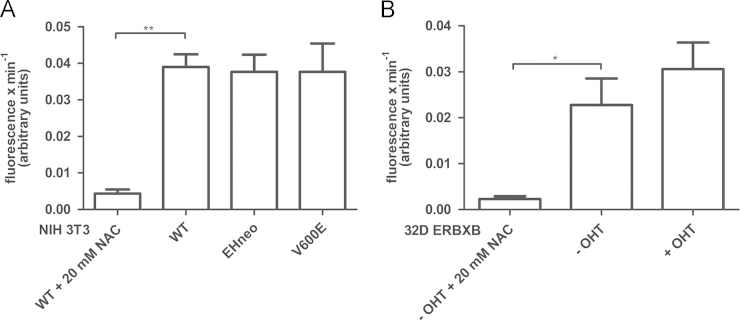
Effect of RAF signaling on the cellular total antioxidant capacity (TAC). (A) NIH 3T3 WT, NIH 3T3 gag-v-RAF (EHneo), NIH 3T3 B-RAF V600E (V600E) and (B) 32D ERBXB cells (+/− OHT) were cultivated for 24 h in the growth media, lysed and subjected to TAC analysis with spectrofluorometer using DCF-DA fluorescent probe upon addition of 20 mM H_2_O_2_. Addition of H_2_O_2_ to NIH 3T3 WT and 32D ERBXB (-OHT) cell lysates in the presence of 20 mM NAC served as a negative control for ROS production. The data are presented as a mean value of changes in the fluorescence per minute (*n*=3, *p≤0.05, **p≤0.01).

Published work demonstrated the ability of survival proteins including RAF, AKT and Bcl-2 to maintain mitochondrial ROS homeostasis [3]. The use on uncoupling reagents had resulted in a decrease in mitochondrial ROS levels during GF starvation [3], suggesting that alterations in mitochondrial ROS levels reflected changes in their production rather than in their detoxification. We also failed to detect differences in the expression of key antioxidant systems [3]. To further corroborate these findings we also compared parental and cells protected by oncogenic RAF for their total antioxidant capacity (TAC), which reflects the ability of the cell to withstand pro-oxidant stress. Cell lysates were prepared as described in the section “Material and methods” and directly tested for their ability to produce ROS. As shown in Fig. 7A and B we failed to detect differences resulting from the expression of oncogenic RAF further supporting the hypothesis that the control of ROS levels by RAF signaling occurs at the level of their production.

## Discussion

Genetic and biochemical evidence as well as the application of kinase inhibitors for the treatment of human tumors have provided evidence for the survival function of RAF kinases [5]. The exact mechanisms remain to be defined. Intriguingly our previous work established a link between the survival activity of RAF and the ability to maintain mitochondrial ROS and Ca^2+^ homeostasis [3]. This may be a more general mechanism, as ROS generation is commonly observed after GF abrogation and other cellular stresses and their accumulation is also counteracted by the survival proteins AKT and Bcl-2. Mitochondrial ROS production therefore may constitute a common endpoint in cell death induction.

In the work presented here we wanted to (i) further study the effect of RAF signaling in preventing apoptotic cell death and excessive mitochondrial ROS levels after GF removal, and (ii) to identify events during the activation of the intrinsic cell death pathway, which are subject to the regulation by ROS. For RAF we have observed an upregulation of Mcl-1 and Bim in cells expressing activated forms of the kinase, which is further enhanced following GF removal. This is in contrast to the effects of AKT also reported previously [4]. Increased Mcl-1 transcription has been observed before in hematopoietic cells due to the activation of the RAF/MEK/ERK as well as PI3K/AKT pathway [19,20]. In our experiments we did not observe any upregulation of Mcl-1 protein in NIH 3T3 fibroblasts expressing active AKT. In contrast to RAF, AKT did not inhibit degradation of Mcl-1 after GF removal. ERK phosphorylation of human Mcl-1 on Thr-163 has been linked to increase in its stability and to a slow-down protein turnover [21], while phosphorylation of Ser-159 by GSK3, activated following shutdown of AKT activity after GF removal, targets Mcl-1 for proteasomal degradation [4]. In contrast to 32D cells, GSK3 phosphorylation was not decreased in NIH 3T3 WT cells upon GF removal (data not shown) suggesting that in fibroblasts AKT/GSK3 signaling is not important for the regulation of Mcl-1 stability.

NIH 3T3 fibroblasts carrying active C-RAF and B-RAF kinases had increased Bim expression in full medium. Moreover, migration of Bim on SDS-PAGE gels was retarded suggesting post-translational modification. Phosphorylation of Bim by ERK results in the dissociation of Bim from Mcl-1 or Bcl-2 [22]. Following GF removal we detected further Bim upregulation but no signs of apoptosis. High Mcl-1 levels simultaneously present in these cells may help to sequester pro-apoptotic Bim. Expression of AKT inhibited Bim upregulation induced by GF removal in NIH 3T3 fibroblasts, which may involve forkhead transcription factors [23]. We also detected upregulation of Bim in 32D cells after IL-3 deprivation, which was suppressed by constitutive or inducible expression of activated C-RAF. C-RAF also promoted changes in Bim mobility on SDS-PAGE gels, but it did not induce its upregulation. The lacking increase in Bim protein after IL-3 abrogation in 32D cells expressing active C-RAF may result in part from PI3K/AKT signaling and its suppression of Bim expression. We also did not detect any changes in the protein level of other Bcl-2 proteins like Puma, Bad, BAK or BAX in NIH 3T3 cells expressing constitutively active C-RAF EHneo or B-RAF V600E (data not shown). No changes were also observed in 32D cells carrying activated C-RAF [3].

Our experiments showed that GF removal resulted in BAX activation in WT cells, which was prevented by active C-RAF or B-RAF in NIH 3T3 fibroblasts and C-RAF in 32D cells. We also detected a striking increase in the mitochondrial content of BAX protein in NIH 3T3 and 32D control cells after GF deprivation. Active C-RAF or B-RAF in NIH 3T3 fibroblasts and C-RAF in 32D cells suppressed BAX translocation to the mitochondria. Interestingly, expression of C-RAF S427G in NIH 3T3 cells and ERBXB in both cell lines maintained in growth medium promoted increase in mitochondrial BAX protein level. Although in healthy cells BAX localized mostly in the cytosol, there are studies showing that a subset of this protein can be loosely attached to the mitochondrial outer membrane. Bcl-2 protects cells from cell death by the inhibition of BAX insertion into the mitochondrial outer membrane, but it also increases loose BAX binding to the outer mitochondrial membrane [24]. We detected upregulation of Mcl-1 in NIH 3T3 and 32D cells carrying active RAF kinases. Mcl-1 may bind to BAX and suppress its proapoptotic activity in these cells. Expression of AKT in NIH 3T3 cells after GF removal prevented BAX activation in these cells and diminished BAX translocation to the mitochondria. AKT was suggested to regulate BAX by direct phosphorylation and thereby inhibiting its translocation to the mitochondria [25].

It has been shown by us that ROS play an important role in cell death induction in 32D cells after GF deprivation [3]. The addition of antioxidant *N*-acetyl-cysteine (NAC) or overexpression of manganese superoxide dismutase (MnSOD) suppressed increase in ROS generation and promoted cell survival [3]. To define the events potentially regulated by endogenously produced ROS in the cells undergoing apoptosis after GF removal we focused on 32D cells. We observed inhibition of caspase-3 activation when 32D cells were starved in the presence of antioxidant NAC resulting in protection against cell death. Moreover, NAC protected 32D cells from a decline in the mitochondrial membrane potential following IL-3 removal. NAC treatment did not exhibit an effect on Bim upregulation but suppressed destabilization of Mcl-1 after IL-3 removal. Interestingly, we did not observe activation of BAX in the presence of NAC in 32D cells under limited GF conditions. However, BAX was translocated to the mitochondria. Activation and translocation of BAX have been pointed out as essential triggers for mitochondrial permeabilization, cytochrome *c* release and subsequent caspase activation. BAX may have to be activated first prior to translocation to the mitochondria [26], whereas others proposed that BAX first is inserted into the outer mitochondrial membrane followed by an activation step [27]. Moreover, the requirement of additional stimuli, like phosphorylation of BAX at the mitochondria by mitogen kinase p38 has been demonstrated [28].

Major effects on intracellular signaling have been described for ROS, in particular following pro-oxidant treatment of cells [29]. However, in our experiments, antioxidants did not prevent changes induced by GF abrogation in the phosphorylation of tested kinases in 32D cells. While our results clearly support a role for ROS in causing cell death, others have seen ROS as a byproduct of the mitochondrial and cellular collapse. BAX deletion inhibited ROS burst and cell death in nerve GF (NGF)-deprived neurons [30]. Other studies demonstrated that BAX presence at the mitochondria is required for ROS-mediated oxidation of cardiolipin and cytochrome *c* release [31]. Our experiments, however, suggest that endogenously generated ROS in the mitochondria after GF deprivation may contribute to BAX activation and loss of mitochondrial membrane potential in 32D cells thereby promoting caspase activation and cell death.

Prosurvival effects of RAF independent of MEK have been reported for mitochondrially located active C-RAF, which phosphorylates Bad promoting its disassociation from Bcl-2 [32,33]. RAF also has been shown to exhibit antiapoptotic function independent of its kinase activity, e.g. through binding to apoptosis signal-regulating kinase (ASK1), suppressing its proapoptotic function [34,35]. Moreover, a study using knock in of mutant C-RAF suggested that MEK kinase activity is not required for C-RAF in normal mice development and protection against apoptosis [36]. However, these data require independent confirmation since the mutant used (YY340/341FF) possesses reduced but detectable kinase activity, has only a moderate effect on C-RAF kinase activity [37].

In terms of maintaining ROS and Ca^2+^ homeostasis RAF and MEK behaved equally in our experiments [3,15]. The data presented here provide further support to such a regulatory mechanism. The MEK inhibitor UO126 abolished the effect of constitutively active C-RAF or B-RAF on Mcl-1 stability or Bim modification after GF removal. BAX activation was also detected in these cells resulting in caspase-3 cleavage. Interestingly, application of PI3K inhibitor LY 294002 abolished C-RAF but not B-RAF antiapoptotic effects, suggesting that C-RAF requires also PI3K activity for cell death inhibition. One possible explanation is that C-RAF-induced autocrine secretion of a GF as shown previously [38].

## Conclusions

Mitochondrial ROS production is essential to turn growth factor (GF) removal into cell death, which was prevented equally well by activated RAF, AKT, Bcl-2 and antioxidants. To investigate whether protection by survival proteins and antioxidants utilizes shared or distinct targets, we used serum deprivation from NIH 3T3 fibroblasts and IL-3 withdrawal from promyeloid 32D cells. Signaling by activated RAF but not AKT prevented the decline in Mcl-1 following GF abrogation. GF starvation increased levels of Bim in both model systems, which was prevented by RAF in 32D cells but not in NIH 3T3 fibroblasts. RAF and AKT suppressed activation and mitochondrial translocation of BAX. Most importantly our data show that antioxidants prevented BAX activation but not mitochondrial translocation and had no significant impact on Bim or Mcl-1 expression. Also ROS produced did not alter the activity of intracellular signaling pathways. Together these data suggest that Bcl-2 family proteins are critical for the survival activity of RAF and antioxidants.

## Disclosure statement

The authors state that they do not have to disclose any actual or potential conflict of interest including any financial, personal or other relationships with other people or organizations within three years of beginning the submitted work that could inappropriately influence, or be perceived to influence, their work.
